# Prmt5 deficiency inhibits CD4+ T-cell Klf2/S1pr1 expression and ameliorates EAE disease

**DOI:** 10.1186/s12974-023-02854-2

**Published:** 2023-08-02

**Authors:** Yingxia Zheng, Zheyi Chen, Bingqian Zhou, Shiyu Chen, Ningdai Chen, Lisong Shen

**Affiliations:** 1 Department of Laboratory Medicine, Xin Hua Hospital, Shanghai Jiao Tong University School of Medicine, Shanghai, China; 2 Faculty of Medical Laboratory Science, Shanghai Jiao Tong University School of Medicine, Shanghai, China; 3 Institute of Artificial Intelligence Medicine, Shanghai Academy of Experimental Medicine, Shanghai, China

**Keywords:** Prmt5, S1pr1, Klf2, Migration, EAE, CD4+ T

## Abstract

**Background:**

Protein arginine methyltransferase 5 (Prmt5) is the main type II methyltransferase, catalyzes protein arginine residue symmetric dimethylation, and modulates normal cellular physiology and disease progression. Prmt5 inhibition or deletion in CD4+ T cells has been reported to ameliorate experimental autoimmune encephalomyelitis (EAE), but the detailed molecular mechanisms have not yet been elucidated.

**Methods:**

EAE was induced by administration of myelin oligodendrocyte glycoprotein (MOG35–55) in T cells Prmt5 conditional knockout (CD4-cre-Prmt5^fl/fl^, Prmt5cko) and Prmt5^fl/fl^ (WT) mice. Flow cytometry, single-cell RNA sequencing, ATAC sequencing and chromatin immunoprecipitation assay (ChIP) approaches were used to explore the detail mechanisms.

**Results:**

We find that Prmt5cko mice are resistant to EAE; infiltrating inflammatory CD4+ T cells in the central nervous system (CNS) are greatly reduced. However, in Prmt5cko mice, T cells in the spleen show much more proliferation and activation properties, the total number of CD4+ T cells in the spleen is not reduced, and the percentage of Rora+ CD4+ T cells is elevated. Also, CD4+ T cells express lower levels of S1pr1 and Klf2 than WT mice, which may influence pathogenic CD4+ T-cell egress from the spleen and migration to the CNS. Moreover, the single-cell ATAC sequence and ChIP assay reveal that the transcription factor Klf2 is enriched at the S1pr1 promoter and that Klf2 motif activity is reduced in Prmt5cko mice.

**Conclusions:**

Our study delineates the undiscovered role of Prmt5 in T-cell biology in which Prmt5 may inhibit Klf2-S1pr1 pathway to ameliorate EAE disease. Controlling T-cell Prmt5 expression may be helpful for the treatment of autoimmune diseases.

**Supplementary Information:**

The online version contains supplementary material available at 10.1186/s12974-023-02854-2.

## Background

Experimental autoimmune encephalomyelitis (EAE) is a perfect model for the study of multiple sclerosis (MS), which is a very common chronic neuroinflammatory disease [1, 2]. Multiple inflammation, axonal demyelination, degeneration and disability are the characteristics of MS. Immune cells, including macrophages, monocytes, dendritic cells, B cells, T cells and natural killer cells, are found in the inflamed central nervous system (CNS) and play important roles in the progression of disease. Activation and expansion of inflammatory Th1 and Th17 cells in the CNS are considered the most important causative factors that relate to MS relapses, drive chronic tissue damage and influence disease severity [3, 4]. Targeting infiltration pathogenic T cells from the periphery has been thought to be an effective therapy for MS [5, 6]. However, how pathogenic T cells migrate to the CNS and the underlying molecular mechanisms remain unclear.

Prmt5 is the type II protein arginine dimethylation transferase that has many roles in cell development and function. It participates in pre-mRNA processing, transcription and DNA damage repair. Full-body deletion of Prmt5 is embryonic lethal, and conditional knockout of Prmt5 results in reduced hematopoietic progenitor cells; moreover, hematopoietic stem and progenitor cells show a decreasing response to cytokine stimulation [7]. Prmt5 is also essential for the maintenance of peripheral T cells by promoting the γ chain and Jak3 pre-mRNA splicing [8]. Recently, our group revealed that T cell-specific deletion of Prmt5 promoted T-cell differentiation into Klrg1+ terminal T cells and promoted cancer progression [9]. The Prmt5-specific inhibitor EPZ015666 suppresses human CD8+ T-cell proliferation and function by inducing P53 expression and reducing the AKT signaling pathway [10]. Methylation is thought to be crucial for the immune response and promotes MS pathogenesis, and inhibitors of panmethylation are associated with relieving EAE clinical scores by impairing Th1 and Th17-cell activity [11]. CNS-infiltrating cells express high levels of Prmt5, which is associated with EAE activity and is correlated with MS peak and relapse by promoting Th1-cell cycle progression [12]. A selective inhibitor of Prmt5 reduces the clinical score of EAE, suggesting the critical role of Prmt5 in autoimmune disease [13]. Moreover, Prmt5 has been reported to be associated with Th1/Th2/Th17/Treg cells polarization [14]. However, whether Prmt5 regulates T-cell migration and affects the severity of EAE is still unknown, and the specific mechanisms need to be further elucidated.

In this study, we used CD4-cre-Prmt5^fl/fl^ conditional knockout (Prmt5cko) mice and demonstrated that Prmt5 is crucial for the pathology of EAE. T cells with specific deletion of Prmt5 showed complete resistant of EAE disease; few pathologic CD4+ T cells infiltrated in the CNS. However, interestingly, we found that after EAE induction, spleens from Prmt5cko mice were enlargement, the number of CD4+ T cells in the Prmt5cko group was not reduced compared with that in the WT. Applying single-cell RNA sequencing to dissect the complexity of cell components in the spleen during the disease peak of EAE, we found that the pathogenic Rora+ CD4+ T-cell cluster was enriched in Prmt5cko mice and those cells expressed lower levels of molecules related to T-cell migration to the CNS. Moreover, highly proliferative CD4+ T cells were observed in vivo and were quite different from those observed in vitro. Furthermore, we applied single-cell ATAC sequencing (scATAC-seq) to probe T-cell functional diversity after Prmt5-specific deletion and to obtain objectively pure transcription profiles for T-cell subsets at the peak of EAE. ChIP assay further revealed that the transcription factor Klf2 was enriched at the S1pr1 promoter and the binding activity was reduced in the Prmt5cko mice. Our analysis elucidated the gene expression signatures and chromatin landscapes of T-cell subpopulations concomitantly and revealed the previously unreported role of Prmt5 in the regulation of T-cell function. Controlling Prmt5 expression in CD4+ T cells may be crucial for regulating CD4+ T-cell function and ameliorating EAE disease.

## Materials and methods

### Animal experiments

T cell-specific Prmt5 deletion (CD4-Cre-Prmt5^fl/fl^, Prmt5cko) mice and CD4-Cre-negative littermate controls (WT) were kept in our laboratory [9]. The animals were maintained in a specific pathogen-free facility of the Shanghai Jiao Tong University School of Medicine, Xin Hua Hospital. This study was performed in accordance with the protocols in the guidelines of Xin Hua Hospital, Institutional Animal Care and Use Committee.

### EAE induction

EAE induction was performed according to a previous study [15]. Briefly, each mouse was subcutaneously immunized with 200 µl inoculum containing 300 µg of MOG33-55 peptide (GL Biochem) in 100 µl phosphate buffered saline (PBS) and 100 µl complete Freund’s adjuvant (CFA) (Sigma) containing 5 mg/ml heat killed H37Ra (Difco Laboratories). All mice were injected intravenously with 200 ng pertussis toxin (Sigma) in PBS twice at 0 and 2 days post-immunization. EAE clinical signs were scored according to the following criteria: 0, normal behavior; 1, limp tail; 2, paraparesis (weakness, incomplete paralysis of one or two hind limbs); 3, paraplegia (complete paralysis of two hind limbs); 4, paraplegia with forelimb weakness or paralysis; and 5, moribund state or death.

### Histopathological analysis

Mice were transcardially perfused with 4% paraformaldehyde, and their spinal cords were dissected and stored in 4% paraformaldehyde overnight. Paraffin-embedded 5 µm spinal cord sections were stained with hematoxylin and eosin (H&E) or Luxol fast blue and examined by light microscopy.

### Isolation of mononuclear cells from mouse CNS tissue

Mononuclear cells were isolated from CNS tissue according to a previous study [15]. Briefly, mice were perfused with 10 ml PBS via the heart to eliminate peripheral blood. Mononuclear cells from the brain and spinal cord were centrifuged by Percoll gradient centrifugation (GE Healthcare). Mononuclear cells at the interface between the 37% and 70% Percoll gradients were collected and washed three times with PBS.

### Flow cytometry and cell sorting

Flow cytometry and cell sorting were performed according to our previous study [9]. Briefly, single-cell suspensions were prepared and surface-stained in FACS buffer (PBS with 2% FBS, 1 mM EDTA, and PBS) with monoclonal antibodies. Cells were stimulated for 5 h with a cell stimulator and inhibitor at 1:500 dilutions (eBioscience) for intracellular cytokine staining. After surface staining, cells were fixed and permeabilized with the Fix/Perm Foxp3 Transcription Factor Staining Buffer Set (eBioscience) and BD Cytofix/Cytoperm buffer (BD Biosciences) following the manufacturer’s instructions, and then stained with monoclonal antibodies. Flow cytometric analysis was performed with a FACS Canto II instrument (BD Bioscience) and FlowJo software (TreeStar). CD45+ immune cells and related T-cell subsets from mouse spleens were sorted with a FACS Aria II Cell Sorter (BD Biosciences); post-sort purity was routinely > 95%. The detailed information of antibodies used in the study is listed in Additional file 2.

### Chemotaxis assays

The migration of mouse splenocytes was analyzed in 6.5 mm Transwell chambers (Corning) with 5.0 μm pore polycarbonate filters. A total of 2 × 10^6^ splenocytes were resuspended in 100 μl of RPMI 1640 medium (Gibco) containing 0.1% fatty acid-free BSA (Absin), penicillin (100 U/ml) (Gibco), and streptomycin (100 mg/ml) (Gibco) and then placed on the top of Transwell inserts. The bottom chambers were filled with 600 μl of the same medium containing 20 nM S1P (Aladdin). Cells that migrated to the bottom chamber after 4 h at 37 °C in a 5% CO2 incubator were counted by flow cytometry. The results are expressed as the fold increase in specific migration over nonspecific migration with control medium. Migration index = (S1P group count)/(control group count).

### Quantitative real-time PCR (QRT-PCR)

Total RNA was isolated from the cells and tissues following the standard TRIzol (Takara) protocol. First-strand cDNA was synthesized from total RNA using RT Master Mix (Takara). The transcription levels were detected using SYBR Green-based RT-PCR performed by the ABI StepOne QRT-PCR Detection System (Life Technologies). The mRNA levels were normalized to those of β-actin. The S1pr1, Prmt5, Klf2, and β-actin primer sequences are shown: S1pr1 forward 5′-ATG GTG TCC ACT AGC ATC CC-3′, reverse 5′-CGA TGT TCA ACT TGC CTG TGT AG-3′; Klf2 forward 5′- GAG CCT ATC TTG CCG TCC TTT-3′, reverse 5′- CAC GTT GTT TAG GTC CTC ATC C-3′, Prmt5 forward 5′- CTG AAT TGC GTC CCC GAA ATA-3′, reverse 5′- AGG TTC CTG AAT GAA CTC CCT-3′, and actin forward 5′- TGT CCA CCT TCC AGC AGA TGT-3′, reverse 5′- AGC TCA GTA ACA GTC CGC CTA G-3′, Irf2 forward 5′-AAT- TCC- AAT- ACG-ATA-CCA-GGG-CT-3′, reverse 5′- GAG-CGG-AGC-ATC-CTT-TTC-CA-3′, Prdm1 forward 5′- GCT- GCT- GGG- CTG- CCT- TTG- GA-3′, reverse 5′- GGA-GAG-GAG-GCC-GTT-CCC-CA-3′.

### Bulk RNA-seq and data analysis

Cells from Prmt5cko or WT mice were sorted by flow cytometry. Bulk RNA-seq and data analysis were performed according to our previous study [9]. Briefly, total RNA was extracted from tissue with TRIzol reagent (Invitrogen). The RNA quality was checked by a Bioanalyzer 2200 (Agilent) and kept at − 80 °C. RNA with RIN > 6.0 was considered suitable for further experiments. Complementary DNA (CdNA) libraries were prepared using NEBNextTM Ultra Directional RNA Multiplex Oligos according to the manufacturer’s instructions. The products were purified and enriched by PCR to create the final cDNA libraries, which were then quantified by an Agilent 2200 instrument. The tagged cDNA libraries were pooled in equal ratios and used for 150 bp paired-end sequencing in a single lane of the Illumina HiSeq X Ten. Before read mapping, clean reads were obtained from the raw reads by removing the adaptor sequences and low-quality reads. The clean reads were then aligned to the mouse genome (GRCm38) using Hisat2. HTseq was used to obtain gene counts, and the RPKM method was used to determine gene expression. The DESeq2 algorithm was applied to filter the differentially expressed genes. Genes were defined as differentially expressed according to the following criteria: fold change > 1.0, *P value* < 0.05, FDR < 0.05.

### Single-cell sequencing and data analysis

Single-cell sequencing was performed according to our previous report [9]. The scRNA-Seq libraries were generated using the 10X Genomics Chromium Controller Instrument and Chromium Single Cell 3’ V3 Reagent Kits (10X Genomics, Pleasanton, CA). All libraries were sequenced by an Illumina sequencer (Illumina, San Diego, CA) in a 150-bp paired-end run. scRNA-seq data analysis was performed by NovelBio Bio-Pharm Technology Co. Ltd.

### Single-cell ATAC-seq

Single-cell ATAC-seq and data analysis were performed by Jiayin Biotechnology Ltd. Briefly, 1*10^6^ spleen cells were isolated from Prmt5cko and WT mice at the peak of EAE. Nuclei were prepared as outlined in the 10 X Genomics Chromium single-cell ATAC-seq solution protocol. Nuclei were loaded with a capture target of 1*10^6^ nuclei per sample. scATAC-seq libraries were prepared for sequencing following the 10X Genomics single-cell ATAC-seq solution protocol. scATAC-seq libraries were sequenced using PE50 sequencing on an Illumina NovaSeq 6000. We then aggregated samples using Cell Ranger-ATAV with the library depth normalization parameters. Downstream analysis was performed using Seurat v4.0.3 and Signac v1.2.1. Filtering parameters for each sample with the following criteria: peak_region_fragments < 1000 and > 40,000, blacklist_ratio < 0.001, and TSS.enrichment > 2. RunTFIDF, with the default parameters being used to normalize each sample. GeneActivity was applied to calculate geneActivity with the following formula: scale.factor = median (nCount_geneActivity of each sample). Then, FindTopFeatures was used to find the top features, with min.cutoff = “q10”. Dimensionality reduction was performed using the SVD with default parameters. FindMarkers was used to filter the difference peak using the following parameters: logfc.threshold = 0, min.pct = 0, test.use = “LR”, and latent.vars = “peak_region_fragments”, and peaks were annotated with ChIPseeker using the following parameters: tssRegion = (−3000, 3000), and genomicAnnotationPriority = c ("Promoter","5UTR", "3UTR","Exon", "Intron", "Downstream", "Intergenic"). To search for a motif, we first used the getMatrixSet function to extract the pfm of the motif from the JASPAR2020 package using the following the parameter: opts = list (tax_group = "vertebrates", all_versions = FALSE). Then, we used CreateMotifMatrix to search for the motif within 150-bp upstream and downstream of the center of the peak and to generate a motif-peak matrix.

### Chromatin immunoprecipitation assay (ChIP assay)

Prmt5cko and WT mice were induced EAE by MOG35–55/CFA immunization. At the peak of day 17, spleens were acquired; CD4+ T cells were isolated by mouse CD4+ T Cell Isolation Kit (Miltenyi). Approximately 5 × 10^6^ CD4+ T cells were cross-linked with 1% formaldehyde for 10 min at 37 ℃ and quenched with 125 mM glycine for 5 min at room temperature. Chromatin was fragmented using Sonics VCX130. Subsequently, the chromatin fragments were precleared and incubated with 5 μg anti-Klf2 Abs (GeneTex, GTX03383), anti-H3k4me3 Abs (Abcam, ab8580), anti-H4R3me2s (Active Motif, 61187) or control IgG (Abcam, ab172730), respectively, at 4 °C overnight. Immunoprecipitated complexes were collected using ChIP Assay Kit (Beyotime). Precipitated DNA was amplified by Q-PCR and normalized to the input. Klf2 promoter region primers were prepared, forward: 5′-ACC AGC TCA CTC GCA AAG TT -3′, reverse: 5′- GCG CTC AGA GAC TTC GTC TT -3′.

### Statistical analysis

Student’s *t* test was used to analyze the differences between two groups. All presented data are the mean ± SEM. Statistical analyses were performed with Prism 7 (GraphPad Software). *P* < 0.05 was considered to indicate a statistically significant difference.

## Results

### CD4-cre-Prmt5^fl/fl^ mice were resistant to EAE

**Fig. 1:**
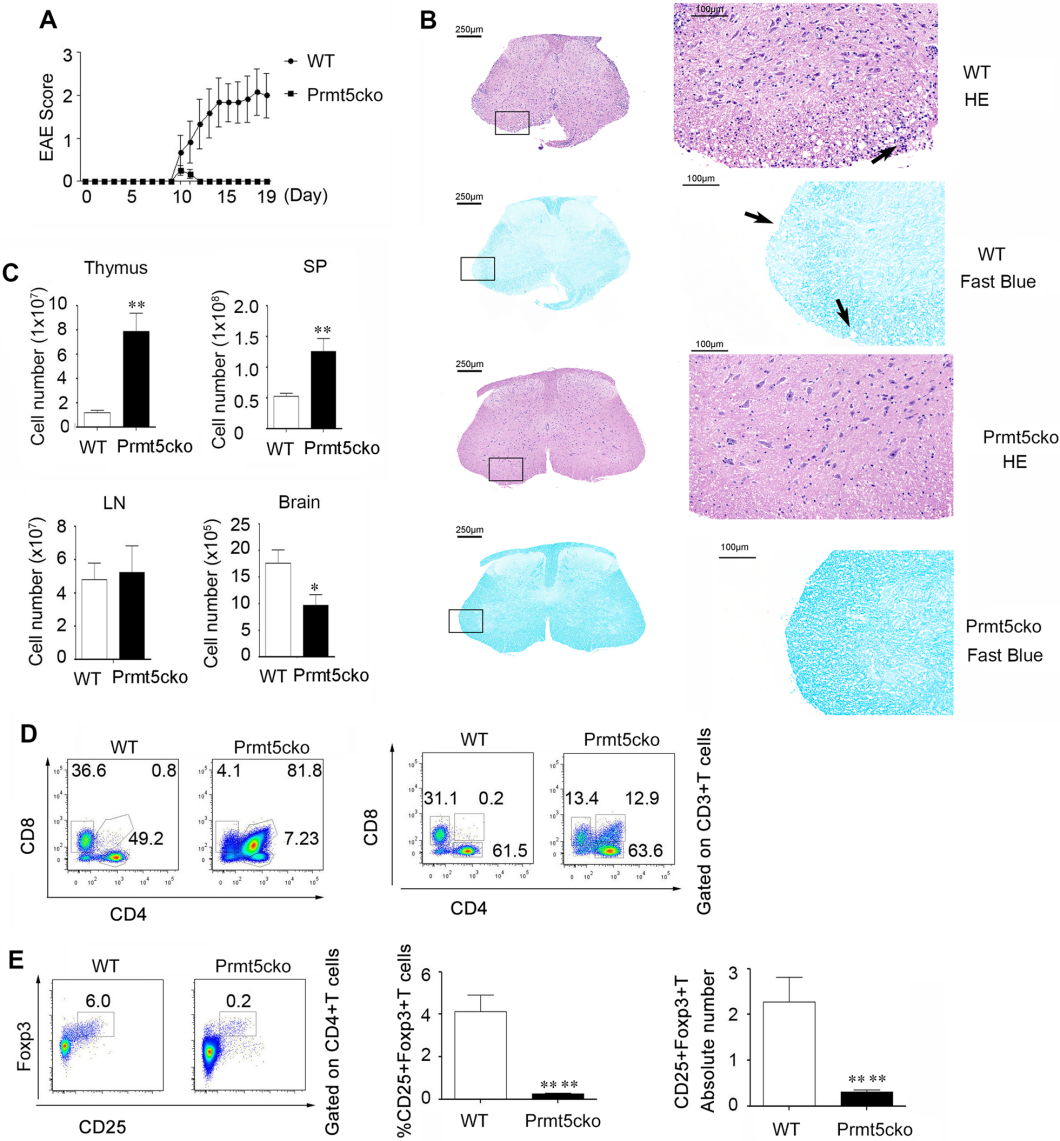
T cell-specific deletion of Prmt5 prevents the induction of EAE autoimmunity.** A** EAE score in Prmt5cko and WT mice after MOG35–55/CFA immunization (6 mice per group), three independent experiments have been performed. **B** Histopathology of spinal cord tissue sections from EAE mice by H&E and fast blue staining at the peak of disease (Day 17), representative images were shown from six mice per group. **C** The absolute mononuclear cell numbers in thymus, spleen, lymph node (LN), and brain tissues from Prmt5cko and WT mice after 17 days of MOG35–55/CFA immunization (6 mice per group), three independent experiments have been performed. **D** CD4 and CD8 expression in the thymus of Prmt5cko and WT mice after MOG35–55/CFA immunization (Day 17). Gated in total thymus cells (left) and CD3+ T cells (right), three independent experiments have been performed. **E** The percentage and absolute number of Tregs in the thymus on Day 17 of EAE induction in Prmt5cko and WT mice, three independent experiments have been performed. Student’s t test. Bar graphs display the mean ± SEM. *P < 0.05, **P < 0.01, ****P < 0.0001

First, we introduced an EAE mouse model to explore the role of Prmt5 in T-cell function. The data showed that WT mice showed significant weight loss when the disease was developed, whereas Prmt5cko mice maintained their weights and had nearly no EAE symptoms (Fig. 1A). We then stained spinal cords with H&E or Luxol fast blue to evaluate the severity of inflammation and demyelination. Spinal cord lesions from WT mice showed many infiltrated immune cells and displayed serious inflammation and demyelination, while Prmt5cko mice showed nearly no inflammation (Fig. 1B). We then isolated thymus, spleen, lymph node (LN) and brain tissues to determine the amount of mononuclear cells. The thymus in the WT mice was shrinking, while it was normal size in the Prmt5cko mice (Additional file 1: Fig. S1A and B). Mononuclear cells in the spleen from Prmt5cko mice were increased, but the infiltrating immune cells in the CNS were significantly reduced; there was no significant difference between these two groups in the LN (Fig. 1C). We then determined the T-cell maturation states in the thymus and found that CD4+ CD8+ immature T cells had nearly disappeared in the WT mice. When gating on CD3+ T cells, the frequencies of CD4+ T cells were similar between these two groups, but CD8+ T cells were greatly decreased in the Prmt5cko group (Fig. 1D). Similar to the naïve mouse thymus, the frequency and absolute number of CD4+ Foxp3+ T cells (Tregs) were reduced in Prmt5cko EAE mice (Fig. 1E) [9]. Collectively, these results suggest that Prmt5 in T cells was critical for EAE induction at both the clinical and histological levels.

### Prmt5 deficiency in T cells showed much more activity in the spleen during EAE induction

**Fig. 2:**
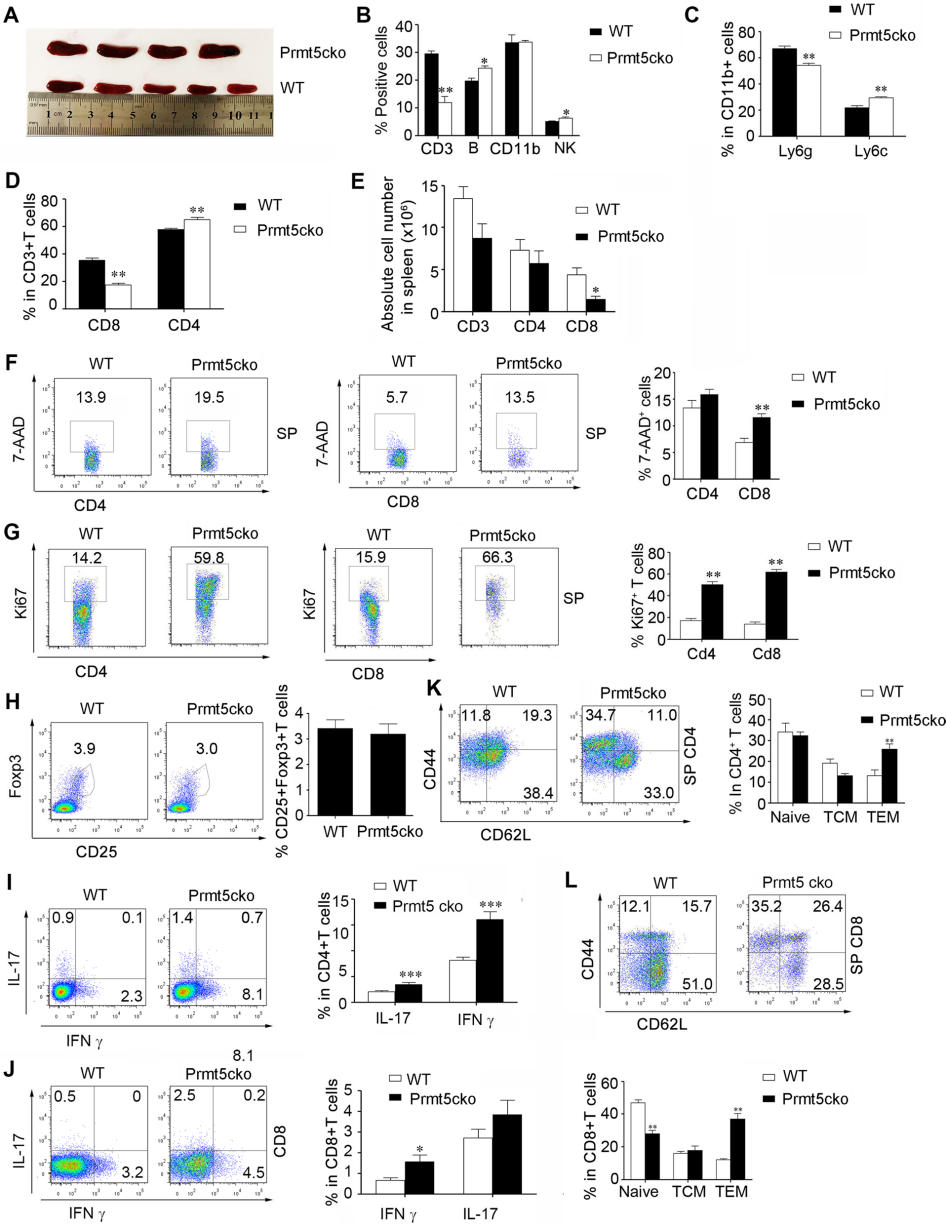
Prmt5 deficiency in T cells showed much more activity in the periphery during EAE. Prmt5cko and WT mice (6 mice per group) were induced EAE by MOG35–55/CFA immunization. At the peak of day 17, spleens were acquired.** A** Graph of spleens between these two groups. **B** The percentages of CD3+ T, B, NK and CD11b+ cells in the two groups. **C** The percentages of CD11b+ Ly6g+ cells and CD11b+ Ly6c+ myeloid cells in two groups. **D** The percentages of CD4+ and CD8+ T cells gated on CD3+ T cells in the two groups. **E** The absolute numbers of CD3+, CD4+ and CD8+ T cells in the two groups. **F** The percentages of dead CD4+ T and CD8+ T cells in the two groups. **G** The percentages of proliferating CD4+ T and CD8+ cells in the two groups. **H** The percentages of Foxp3+ CD25+ Tregs in CD4+ T cells between the two groups. **I** The percentages of CD4+ IL-17+ and CD4+ IFNγ + T cells in the two groups. **J** The percentages of CD8+ IL-17+ and CD8+ IFNγ+ T cells in the two groups. **K, L** The percentages of CD62L+ CD44− (naïve), CD62L+ CD44+ (CM), and CD62L−CD44+ (EM) T-cell subsets of CD4+ T (**K**), CD8+ T (**L**) cells in the two groups. Data are shown as the mean ± SEM of indicated number of samples and are from a single experiment representative of three experiments performed. Statistical differences were determined by the two-tailed unpaired Student’s *t* test, *P < 0.05, **P < 0.01, ***P < 0.001

Although EAE disease severity was ameliorated in Prmt5cko mice, spleens were enlargement and were much bigger than those in the WT group (Fig. 2A, Additional file 1: Fig. S1C). The frequency of CD3+ T cells was reduced, while those of B cells and NK cells were increased in the Prmt5cko mice (Fig. 2B). Although CD11b+ myeloid cells showed no difference between these two groups, Ly6c+ myeloid cells were increased and Ly6g+ neutrophils were reduced in Prmt5cko mice (Fig. 2B and C). The frequency of CD4+ T cells was elevated, while that of CD8+ T cells was reduced, and the absolute number of CD4+ T cells showed no significant difference between these two groups (Fig. 2D and 2E). Apoptosis of CD8+ T cells were increased in the spleen and lymph nodes of Prmt5cko mice (Fig. 2F, Additional file 1: Fig. S2A). Surprisingly, the proliferation of both CD4+ and CD8+ T cells was greatly elevated in Prmt5cko mice compared with WT mice (Fig. 2G and Additional file 1: Fig. S2B), which was quite different from the in vitro data showing impaired proliferation (Additional file 1: Fig. S2C), suggesting that in the EAE microenvironment, Prmt5cko mice may have some factors that stimulate T-cell proliferation. Interestingly, while CD4+ Tregs from the spleen showed no differences between these two groups, Tregs from both CD4+ and CD8+ T cell populations were elevated in LN tissue in Prmt5cko mice (Fig. 2H and Additional file 1: Fig. S2D). Moreover, CD4+ T cells produced higher levels of IFNγ and IL-17 in the spleen, while there were no significant differences in LN tissue. However, CD8+ T cells from LN tissue presented increasing levels of IFNγ and IL-17 in Prmt5cko mice (Fig. 2I, J, Additional file 1: Fig. S2E). In addition, CD44+ CD62L+ effector memory T cells were significantly enriched in the splenic CD4+ T cell population, and effector memory T cells were enriched in both CD4+ and CD8+ T cell populations in LN tissue (Fig. 2K, L, Additional file 1: Fig. S2F, G). These data suggested that in the EAE inflammatory microenvironment, as in the naïve and cancer microenvironment [9], higher percentages of IFNγ and IL-17 producing CD4+ T cells in the spleen in the CD4-cre-Prmt5^fl/fl^ mice.

### CD4+ T cells from the CNS showed moderate inflammation in Prmt5cko mice

**Fig. 3:**
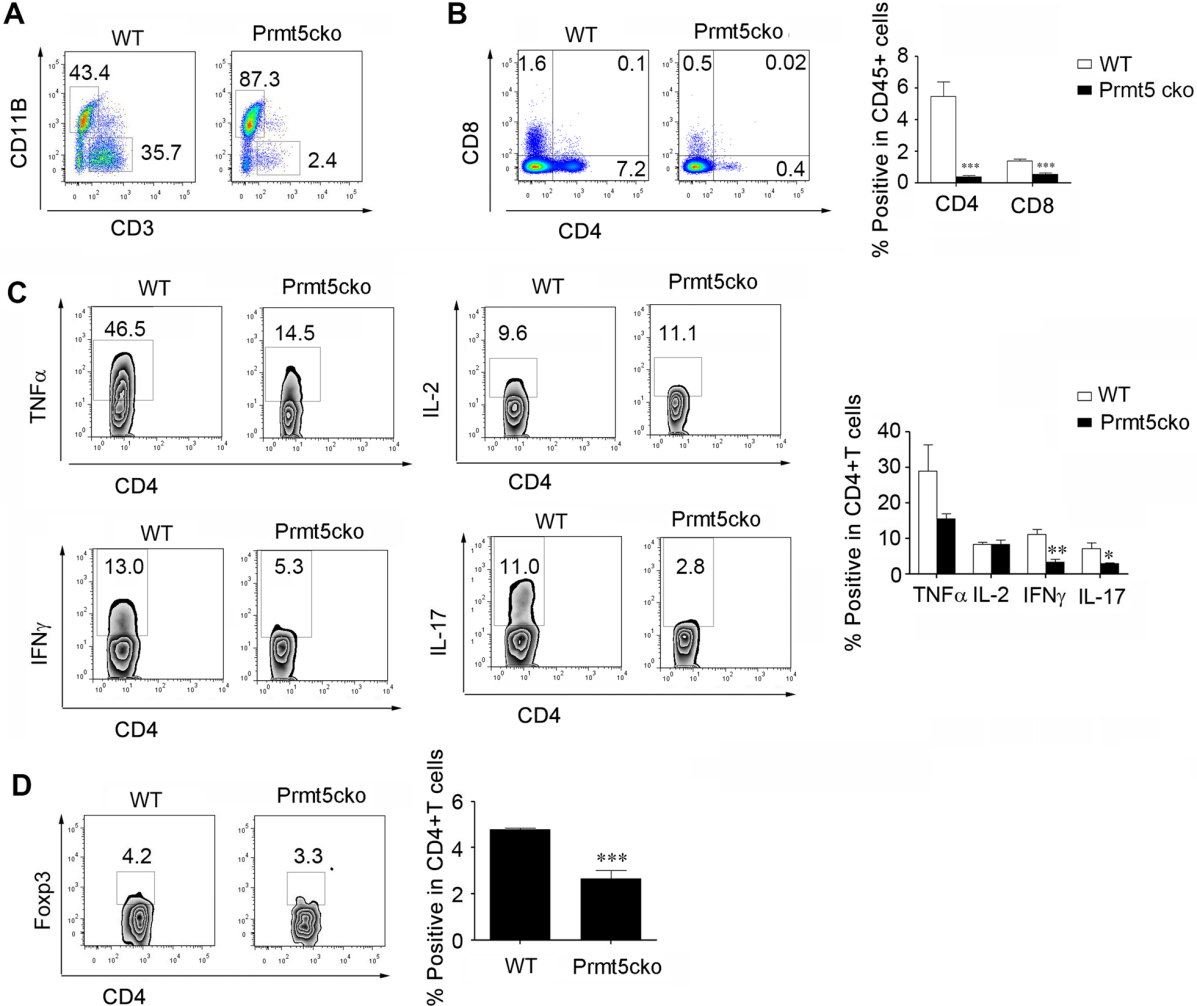
Infiltrating pathogenic CD4+ T cells in the CNS were decreased in Prmt5cko mice after induction of EAE. EAE was induced in Prmt5cko and WT mice (6 mice per group) by MOG35–55/CFA immunization. At the peak of Day 17, brain and spinal cords were acquired. **A** The percentage of CD3+ T cells in the two groups. **B** The percentages of CD4+ and CD8+ T cells in the two groups. **C** CD4+ T-cell expression of IL-17, IFNγ, IL-2 and TNFα was determined. **D** The percentage of Tregs was determined. Data are shown as the mean ± SEM of indicated number of samples and are from a single experiment representative of three experiments performed. Statistical differences were determined by the two-tailed unpaired Student’s t test, *P < 0.05, **P <0.01, ***P<0.001

We further measured infiltrating immune cell subsets from the CNS. The data showed that the percentage of CD3+ T cells was greatly decreased in the Prmt5cko mice (Fig. 3A); in contrast, in the spleen, the percentage of CD4+ T cells was significantly decreased (Fig. 3B). Moreover, CD4+ T cells that produced IFNγ and IL-17 inflammatory cytokines were significantly decreased in Prmtc5cko mice compared with WT mice (Fig. 3C). In addition, Tregs were reduced in Prmt5cko mice (Fig. 3D). These data suggest that infiltrating CD4+ T cells in the CNS from Prmt5cko mice were greatly decreased, consistent with the clinical and histological levels.

### CD4+ and CD8+ T cells from Prmt5cko EAE mice possessed different migration abilities

**Fig. 4:**
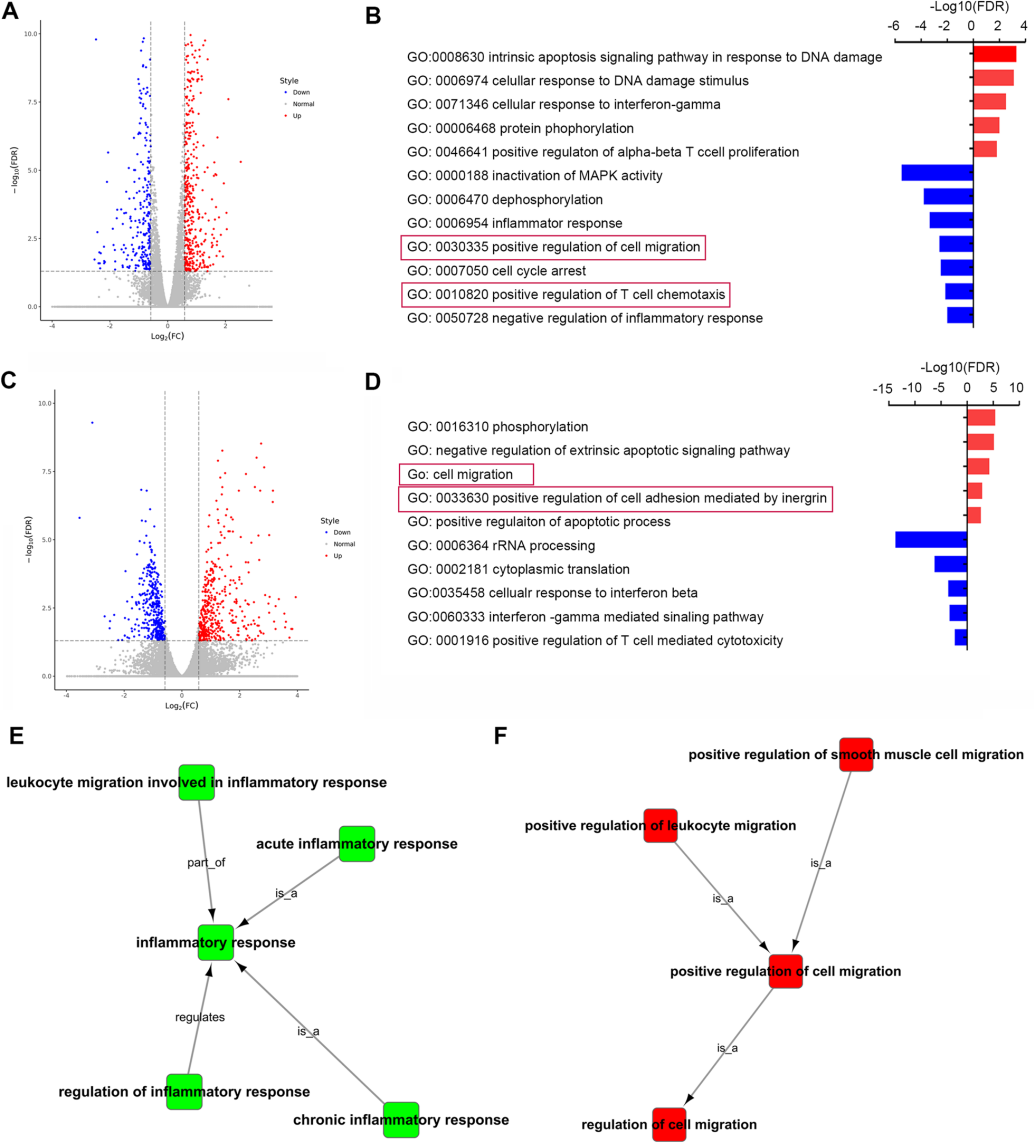
CD4+ and CD8+ T cells from Prmt5cko EAE mouse spleens showed different migration ability. Prmt5cko and WT mice (3 mice per group) were induced EAE by MOG35–55/CFA immunization. At the peak of Day 17, CD8+ and CD4+ T cells were isolated from the spleen, bulk RNA-seq was performed (each mice was constructed library separately). **A** Volcano plot showing the up- and down-regulated genes in CD4+ T cells between WT and Prmt5cko mice, the differentially expressed genes (log_2_FC > 1.0; FDR < 0.05). **B** Bar graph of enriched terms of the differentially expressed genes in CD4+ T cells between the WT and Prmt5cko mice. Red boxes show the GO terms of interest. **C** Volcano plot showing the up- and down-regulated genes in CD8+ T cells between WT and Prmt5cko mice, the differentially expressed genes (log_2_FC > 1.0; FDR < 0.05). **D** Bar graph of enriched terms of the differentially expressed genes in CD8+ T cells between the WT and Prmt5cko mice. Red boxes show the GO terms of interest. **E** GO tree analysis of the differential gene expression in CD4+ T cells showed that low leukocyte migration, which is involved in the inflammatory response, was enriched. **F** GO tree analysis of the differential gene expression in CD8+ T cells showed that positive regulation of cell migration was enriched

The absolute number of CD4+ T cells from the spleen was not decreased in Prmt5cko mice, but the infiltrating CD4+ T cells in the brain were significantly reduced. We then sought to explore the underlying mechanisms. First, we isolated spleen CD4+ T and CD8+ T cells from Prmt5cko and WT mice at the peak of EAE and analyzed their RNA profiles. The number of differentially expressed genes (upregulation and downregulation) in CD4+ T cells was 441 vs*.* 275, respectively, (log_2_FC > 1, FDR < 0.05), while that in CD8+ T cells was 430 vs. 396, respectively, (log_2_FC > 1, FDR < 0.05) (Fig. 4A and C), suggesting the important role of Prmt5 in modulating gene transcriptional repression. Furthermore, Gene Ontology (GO) analysis of the differential gene expression profiles showed that CD4+ T cells from Prmt5cko mice were significantly enriched in cell proliferation but downregulated in the T-cell migration pathway (Fig. 4B). However, GO analysis of the CD8+ T-cell differential gene expression profiles showed an enrichment of positive regulation of cell adhesion by integrin and migration (Fig. 4D). GO tree analysis further suggested that CD4+ T cells from Prmt5cko mice had downregulated genes in leukocyte migration, which is involved in the inflammatory response, but CD8+ T cells had upregulated genes in cell migration (Fig. 4E and F). These data demonstrated that CD4+ and CD8+ T cells potentially had different migration abilities when Prmt5 was deficient. The results may partly explain why CD4+ T cells were decreased in the CNS but not in the spleen. The migration ability of CD8+ T cells was increased; however, the number of apoptotic cells rose at the same time, leading to reduced CD8+ T-cell infiltration.

### Single-cell RNA sequencing revealed that Rora+ CD4+ T cells were enriched in Prmt5cko mice and that CD4+ T cells expressed lower levels of S1pr1

**Fig. 5:**
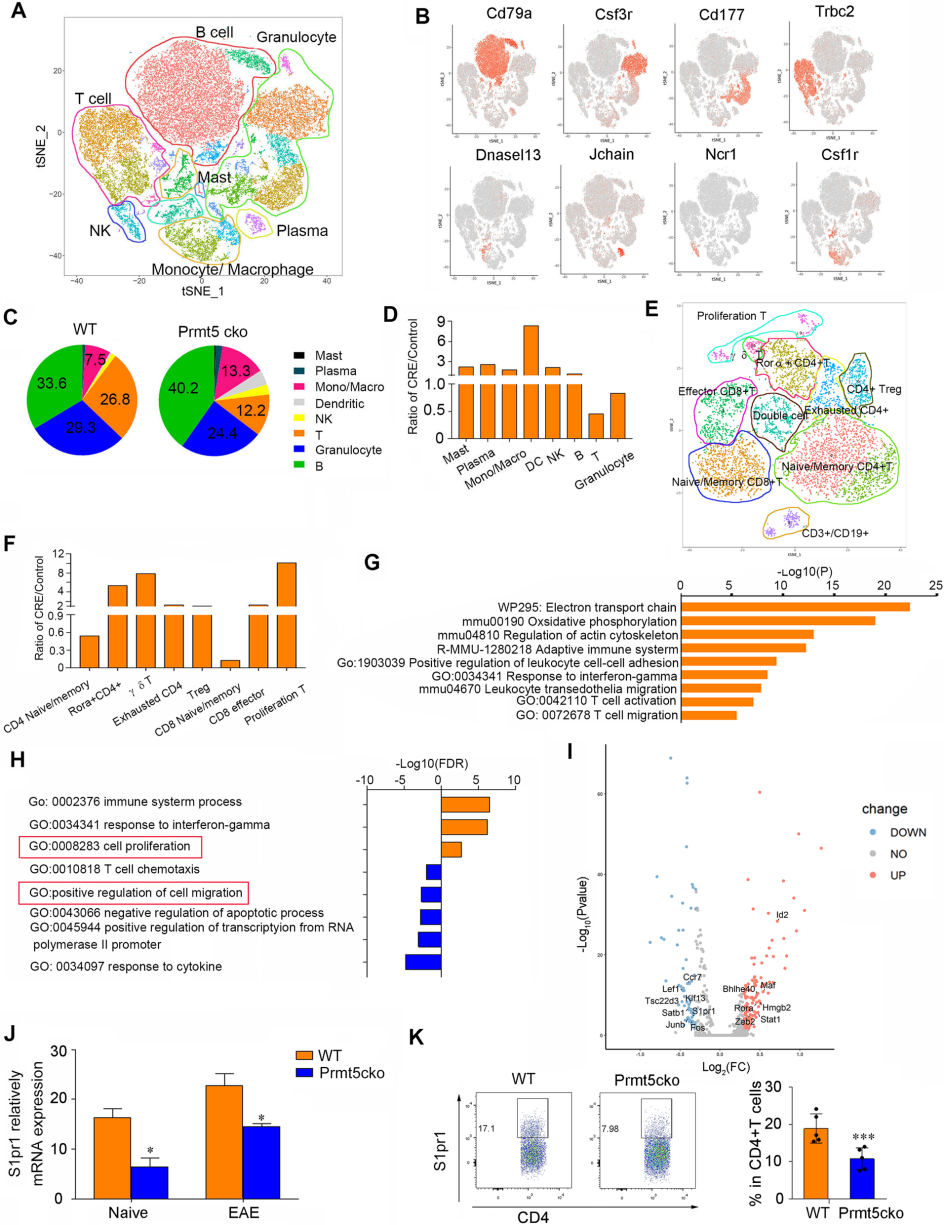
Single-cell RNA sequencing revealed that Rora+ CD4+ T cells were enriched in Prmt5cko mice and had the characteristic of low migration. EAE was induced in Prmt5cko and WT mice (3 mice per group) by MOG35–55/CFA immunization, and at the peak of Day 17, spleen cells were isolated, and single-cell RNA sequencing was performed. **A** tSNE plot showing the spleen cell clusters. Each dot corresponds to a single cell, colored according to the cell cluster. **B** Expression levels of relative marker genes illustrated as tSNE plots. The expression was measured as the log_2_(count + 1). **C** Pie charts of cell-type fractions between the two groups, colored by cell type. **D** The ratios of immune cell subsets between WT and Prmt5cko mice spleens. **E** tSNE plot showing the T-cell reclusters. **F** The ratios of T-cell subsets between Prmt5cko and WT mice. **G** Bar graph showed enriched terms of the marker genes of the Rora+ CD4+ T-cell cluster. Red boxes showing the GO terms of interest. **H** Bar graph showing the enriched terms of the differentially expressed genes of the Rora+ CD4+ T-cell cluster between WT and Prmt5cko mice. Red boxes showed the GO terms of interest. **I** Volcano plot showed the up- and downregulated genes in CD4+ T cells between WT and Prmt5cko mice. **J** S1pr1 expression was measured by RT-qPCR in CD4+ T cells isolated from Prmt5cko and WT mice in the naïve and EAE states. **K** S1pr1 expression was measured in CD4+ T cells from Prmt5cko and WT mice in the EAE condition by flow cytometry (*N* = 5 per group). Data are shown as the mean ± SEM of indicated number of samples and are from a single experiment representative of three experiments performed. Statistical differences were determined by the two-tailed unpaired Student’s *t* test, *P < 0.05, ***P < 0.001

To further explore and dissect the heterogeneities of spleen cells between these two groups in detail, we applied single-cell RNA-seq. We obtained spleen cells from WT and Prmt5cko mice for sequencing. Unsupervised clustering showed that cells were clustered into 24 clusters, including B cells, T cells, NK cells, monocytes/macrophages, DCs, mast cells and neutrophils (Fig. 5A and B and Additional file 1: Fig. S3A). The percentages of B, NK, and monocyte/macrophage and DC cells were increased, while the percentages of T cells, granulocyte cells were decreased in Prmt5cko mice (Fig. 5C and D). We then reclustered T cells on the basis of distinct transcription profiles. T cells were clustered into eight major subsets, including naïve/memory CD4+ T cells, Tregs, exhausted CD4+ T cells, Rora+ CD4+ T cells, naïve/memory CD8 + T cells, effector CD8 + T cells, proliferating T cells and γδ T cells (Fig. 5E, Additional file 1: Fig. S3B). Naïve/memory CD4+ and CD8+ T cells were decreased while the proportions of proliferating T cells and γδ T cells were greatly increased in the Prmt5cko mice compared with the WT mice, and the frequency of CD4+ Tregs showed a slight decrease (Fig. 5F). However, the Rora+ CD4+ T-cell cluster was elevated in Prmt5cko mice (Fig. 5F). Indeed, the expression of Rora+ CD4+ T-cell-related genes, such as Cxcr6, Il18r1, Rora, and Bhlhe40, were higher in this cluster (Additional file 1: Fig. S2B). Metascape analysis showed that this cluster was enriched in T-cell activation and T-cell migration, suggesting the high inflammatory property of this cluster (Fig. 5G). Furthermore, GO analysis of the different genes in the Rora+ CD4+ T-cell cluster between these two groups revealed an upregulation of cell proliferation and downregulation of cell migration in Prmt5cko mice (Fig. 5H). A volcano plot showed the upregulated and downregulated genes between Prmt5cko and WT mouse CD4+ T cells. We observed that S1pr1, which is related to T-cell migration from the periphery to inflammatory tissue, was expressed at lower levels in Prmt5cko mice (Fig. 5I). S1pr1 has been reported to be associated with lymphocyte cell trafficking; for example, it has been shown to regulate CD4+ T-cell egress from the spleen to sites of inflammation [16]. We verified S1pr1 expression in CD4+ T cells from Prmt5cko and WT mice, data showed that S1pr1 expression was downregulated in the CD4+ T cells of Prmt5cko mice (Fig. 5J and K). These data further support that pathogenic T cells were blocked in the spleen and contributed to spleen enlargement, resulting in a dramatic reduction in infiltrating T cells into the CNS and thereby ameliorating the CNS inflammatory response.

### scATAC-Seq revealed Klf2 regulated S1pr1 expression

**Fig. 6:**
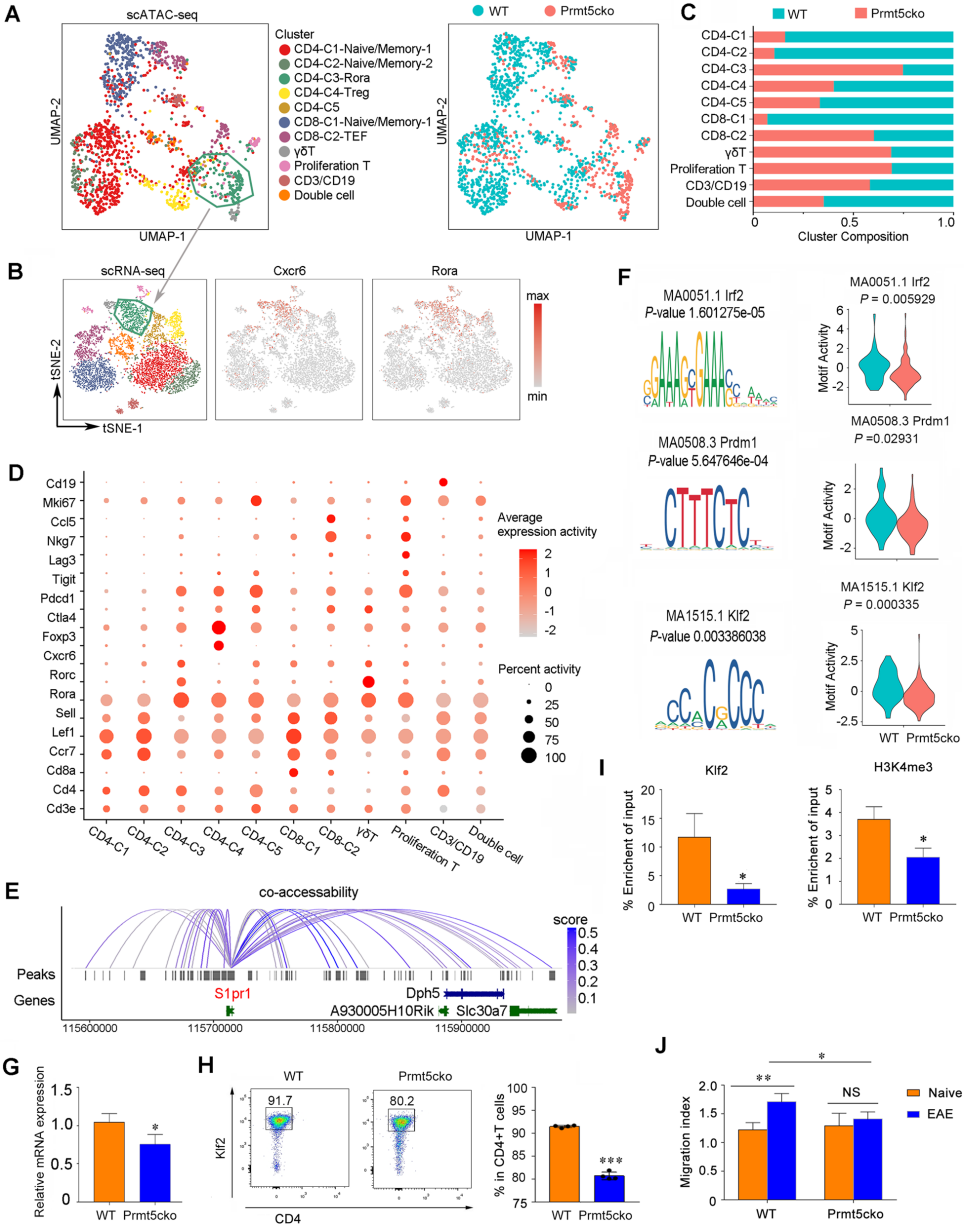
Single-cell analysis of chromatin accessibility associated with T cells under the EAE condition. EAE was induced in Prmt5cko and WT mice (3 mice per group) by MOG35–55/CFA immunization. At the peak of Day 17, spleen cells were isolated, and single-cell ATAC sequencing was performed.** A** scATAC-seq clusters of 1541 T cells pooled from WT and Prmt5cko mice (*n* = 3 each group). **B** Integration of scRNA-based classifications into scTATC data as subpopulation annotations. **C** Cluster composition of scATAC-seq clusters between WT and Prmt5cko mouse T cells. **D** Summary of gene expression activity used to identify T-cell clusters. **E** A set of peaks was identified in all clusters surrounding S1pr1, and 27 peaks showed coaccessibility with the S1pr1 promoter (coaccessibility score > 0.05) via Cicero. **F** Motif analysis and violin plots show the difference of Irf2, Prdm1, and Klf2 motif activities in CD4-C3-Rora cells between the Prmt5cko and WT mice. **G** Klf2 expression was measured by RT-qPCR in CD4+ T cells isolated from Prmt5cko and WT mice in the EAE states (*N* = 3 per group). **H** Klf2 expression was measured in CD4+ T cells from Prmt5cko and WT mice under the EAE condition by flow cytometry (*N* = 4 per group). **I** Enrichment of Klf2 and H3K4me3 at the S1pr1 promoter were assessed by ChIP Q-PCR in CD4+ T cells isolated from Prmt5cko and WT groups in the EAE condition. Data are representative of two independent experiments. Values are presented as the mean ± SEM. **J** Splenocytes were acquired from WT and Prmt5cko mice (*N* = 3 per group) with or without the EAE induction, and CD3+ CD4+ T cells migration ability was stimulated with S1P and the cells numbers were counted by flow cytometry, the migration index = (S1P group count)/(control group count) was calculated between the groups. Data are shown as the mean ± SEM of indicated number of samples and are from a single experiment representative of three experiments performed. Statistical differences were determined by the two-tailed unpaired Student’s t test, *P < 0.05, **P < 0.01, ***P < 0.001

Analysis of chromatin status provides insights into the capabilities of transcription factors (TFs) that shape subset specification and regulate gene expression. We then explored the gene regulatory programs of immune cell populations between Prmt5cko and WT mice at the peak of EAE. We performed scATAC-seq and profiled 7518 CD45+ splenocytes, and we identified the main immune cell subclusters (Additional file 1: Fig. S4A). scATAC-seq peak annotations demonstrated that these clusters reasonably matched the transcriptional signatures of the main immune cell populations identified in scRNA-seq data (Additional file 1: Fig. S4B and C). Moreover, we reclustered T cells and found that these clusters also reasonably matched the single-cell RNA sequencing data (Fig. 6A and B). The proportions of the cell clusters were consistent with the scRNA-seq transcriptional data, and CD4-C3-Rora cells, γδ T cells and proliferation clusters were profoundly expanded in Prmt5cko mice compared to WT mice (Fig. 6C). scATAC-seq further revealed cluster-specific accessibility at cell-specific loci. For example, the Rora and Rorc promoters were more accessible in the CD4-C3-Rora and γδ T clusters than in the other CD4+ T-cell clusters (Fig. 6D). Similarly, Foxp3 promoters were more accessible in the CD4-C4 cluster, suggesting that this cluster was composed of CD4+ Tregs, and proliferating T cells showed epigenetic accessibility at the promoter of highly expressed Mki67 (Fig. 6D). GO enrichment analysis of the significantly downregulated peaks of CD4-C3-Rora cells between Prmt5cko and WT mice showed that these peaks were enriched in regulating cell activation, cytokine production and cell‒cell adhesion (Additional file 1: Fig. S4D). As S1pr1 expression was found to be decreased in the Prmt5cko mice, we then explored the potential transcription factor that regulates S1pr1 expression. We identified a set of peaks surrounding S1pr1, and 27 peaks showed coaccessibility with the S1pr1 promoter (coaccessibility score > 0.05) via Cicero (Fig. 6E). Motif sequence analysis revealed that Irf2, Prdm1 and Klf2 may regulate S1pr1 expression, and violin plots showed that Irf2, Prdm1, and Klf2 motif activities were reduced in CD4-C3-Rora cells between the Prmt5cko and WT mice (Fig. 6F). We then determined Klf2, Prdm1 and Irf2 mRNA expression, data showed that Klf2 expression was significantly decreased while Irf2 and Prdm1 showed not significantly difference between the two groups (Fig. 6G and Additional file 1: Fig. S5A). Klf2 has been reported to promote S1pr1 expression and is critical for T-cell trafficking lymphocyte egress and tissue-resident memory CD8+ T-cell generation [17–19]. Indeed, we further determined Klf2 protein expression by flow cytometry, and found the expression was decreased in Prmt5cko CD4+ T cells (Fig. 6H). Furthermore, ChIP PCR assay showed that Klf2 binding to the S1pr1 promoter was reduced in the Prmt5cko group than the WT group; moreover, the transcriptional activation mark H3K4me3 binding to the S1pr1 promoter was reduced in the Prmt5cko group while transcriptional inhibitory mark H4R3me2s binding showed not difference between the two groups (Fig. 6I and Additional file 1: Fig. S5B). In addition, we found that CD4+ T cells from the Prmt5cko group showed less response to the chemoattractant of S1p than the WT CD4+ T cells in the EAE condition, while CD8+ T cells showed not quite difference in vitro (Fig. 6J and Additional file 1: Fig. S5C). Our results suggested that Prmt5 may regulate CD4+ T-cell function through Klf2/S1pr1 expression and have an effect on EAE progress.

## Discussion

Several studies have reported that Prmt5 is essential for peripheral T-cell development, and selective Prmt5 inhibitors, including CMP5, EPZ015666 and HLCL65, suppress T-cell receptor-induced proliferation of CD4+ and CD8+ T cells [10, 13, 20]. Prmt5 constitutive ablation in mice is embryonic lethal [21], and hematopoietic stem and progenitor cells from bone marrow were dramatically reduced in Prmt5^fl/fl^-Mx1-cre homozygous mice [7]. We and other two laboratories reported that conditional deletion of Prmt5 in T cells results in severely impaired peripheral T-cell compartments, including iNKT, CD4+ and CD8+ T cells, although the findings regarding T-cell maturation in the thymus were different [8, 9, 14]. Webb et al. and colleagues reported that short-term tamoxifen-treated CD4creERT2Prmt5^fl/fl^ mice were completely resistant to EAE development due to the loss of infiltrating MOG-specific pathogenic Th17 and Th1 cells in the CNS, Prmt5 deficiency suppressed T cell proliferation [14], and our results were consistent with these phenotypes. They also reported that T-cell deficiency of Prmt5 resulted in impaired cholesterol metabolism and Ror-γt activation, leading to a severe defect in the differentiation of naïve T cells toward the Th17 phenotype in the in vitro Th cells polarization system, they also found that MOG-specific Th1 and Th17 responses were notably reduced among splenocytes, whereas T-bet+ IL-17+ Th17 cell responses did not reach statistical significance. Interestingly, we had some different findings. While the infiltrating CD4+ T cells, Th17 and Th1 cells in the CNS were indeed consistent, the in vivo data about pathogenic Th17 and Th1 cells in the spleen were different. We found that spleens were enlarged in the Prmt5cko mice during the induction of EAE, and T cells in the spleen showed much more proliferation and activation. The percentages of Th17 and Th1 cells in the spleen were elevated compared with those in the WT mice; the percentage of Ki67+ T cells was elevated. This difference may be due to that we detected the IL-17 and IFNγ expressions in CD4+ T cells under the stimulation with a cell stimulator and inhibitor cocktail containing PMA/inomycin/brefeldin A/monensin for 5 h; while Webb lab used MOG peptide to restimulation splenocytes in vitro. T cells deficiency of Prmt5 showed less response to the cytokines stimulation, which may probably reduce the T cells proliferation and cytokines production in vitro. However, the decreasing percentages of Th17 and Th1 cells in the spleen in the WT mice may have other causes, one explanation would be that effector cells in WT mice reside in the CNS, while Prmt5cko mice effector cells are still in the spleen.

Our data suggested that the reduction in infiltrating pathogenic T cells in the CNS was not due to the loss of pathogenic CD4+ T cells in the periphery but maybe the decreased migration ability of these cells. Indeed, GO analysis of the differentially expressed genes of CD4+ T cells and the in vitro migration assay of the spleens between these two groups revealed that the CD4+ T-cell showed a lower response to S1p in the Prmt5cko mice. scRNA-seq further revealed that CD4+ T cells expressed lower levels of S1pr1, which is important for T-cell egress from the spleen and migration to the CNS. Targeting S1pr1 has been an exciting advancement in MS therapy [22, 23]. Finally, we examined the epigenetic landscape of T cells and identified the gene activity between T cell clusters. Motif analysis revealed that Klf2 was enriched at the S1pr1 promoter; furthermore, Klf2 activities were downregulated in Prmt5cko mice, suggesting that Prmt5 may regulate Klf2 activities and affect S1pr1 expression in Rora+ CD4+ T cells, leading to the compromised migration of this cluster.

Prmt5 has been reported to be associated with the development of multiple cancers, such as lung cancer, cervical cancer, glioblastoma and liver cancer, where it boosts cancer cell hyperproliferation [24–27]. Prmt5 also regulates the E2F pathway, enhances cancer cell migration and invasion, and modulates fibroblast-like synoviocytes in rheumatoid arthritis [28, 29]. To our knowledge, how Prmt5 regulates T-cell migration has not yet been fully clarified. We verified that peripheral CD4+ T-cell Prmt5 expression was an essential driver for the onset and development of EAE disease, and we addressed the different mechanisms. Through in vivo and in vitro study, we found that Prmt5 modulated the peripheral homeostasis stages of T cells, including survival, proliferation, apoptosis and somehow migration. In the absence of Prmt5, CD4+ T-cell showed lower response to S1p may led to a reduction in CNS-infiltrating pathogenic CD4+ T cells. The role of Prmt5 as a cancer driver has been well studied, and Prmt5 inhibitors to treat cancers are currently being assessed in clinical trials [30]. Our study further demonstrated the novel function of Prmt5 in regulating CD4+ T cells function, and this may help to ameliorate MS diseases.

In summary, by integrating single-cell transcriptomics and epigenomics analysis, we addressed the unreported role of Prmt5, which inhibit Klf2-S1pr1 pathway and may account for EAE resistance in Prmt5cko mice. However, our study has limitations, we did not use an original approach to study the roles of Prmt5 in T-cell physiology and homeostasis, also, future studies are required to confirm the critical signals that trigger the development and expansion of Rora+ CD4+ T cells in vivo under EAE conditions; moreover, the role of Prmt5 in CD4+ T cells migration has not been clearly established and warrants further experiments.

## Supplementary Information

**Additional file 1.** Supplementary figure.

**Additional file 2.** Materials information.

## Data Availability

RAN-seq, single-cell RNA-seq and single-cell ATAC-seq raw data to the GEO repository, GEO accession number is GSE195888. Other datasets used during the current study are available from the corresponding author on reasonable request.
