# Supplementary material for: Prmt5 deficiency inhibits CD4+ T-cell Klf2/S1pr1 expression and ameliorates EAE disease
Source: J Neuroinflammation. 2023 Aug 2;20:183. doi: 10.1186/s12974-023-02854-2 (PMC10398933; doi:10.1186/s12974-023-02854-2)
Supplement: Supplementary file 1 — Additional file 1. Supplementary figure. [file 12974_2023_2854_MOESM1_ESM.pdf]

Prmt5 deficiency inhibits CD4+ T-cell Klf2/S1pr1 expression and ameliorates EAE disease

Yingxia Zheng [1, 2, 3, #,*], Zheyi Chen[1, 2, 3,#], Bingqian Zhou[1, 2, 3], Shiyu Chen[1, 2, 3], Ningdai Chen[1, 2, 3], Lisong Shen[1,2,3,*]

[1] Department of Laboratory Medicine, Xin Hua Hospital, Shanghai Jiao Tong University School of Medicine, Shanghai, China;

[2] Faculty of Medical Laboratory Science, Shanghai Jiao Tong University School of Medicine, Shanghai, China.

[3] Institute of Artificial Intelligence Medicine, Shanghai Academy of Experimental Medicine，Shanghai, China.

[#] These authors contributed equally to this paper.

[*]Correspondence e-mails: lisongshen@hotmail.com (L.S.); zhengyingxia@xinhuamed.com.cn (Y.Z.).

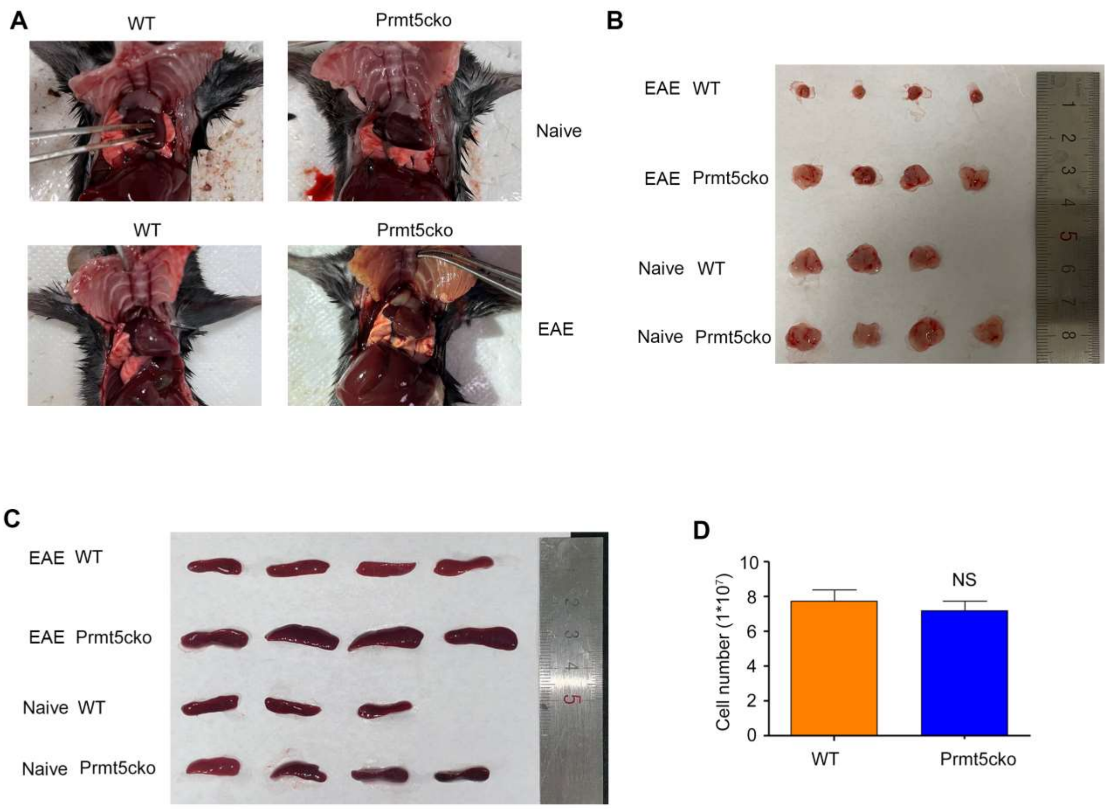

Fig. S1. T cells specific deficiency of Prmt5 ameliorates EAE disease. Prmt5cko and WT mice (4 mice per group) were induced EAE by MOG35－55/CFA immunization, at the peak of day 17, thymus and spleen were acquired. (**A, B, C**) Photographs of thymus (**A, B**) and spleens (**C**) between naïve and EAE control and Prmt5cko mice were shown. (**D**) The absolute mononuclear cell numbers in spleen from WT and Prmt5cko mice in naïve state (N=5). Data are shown as the mean ± SEM of indicated number of samples and are from a single experiment representative of three experiments performed. Statistical differences were determined by the two-tailed unpaired Student's t test, NS: not significant.

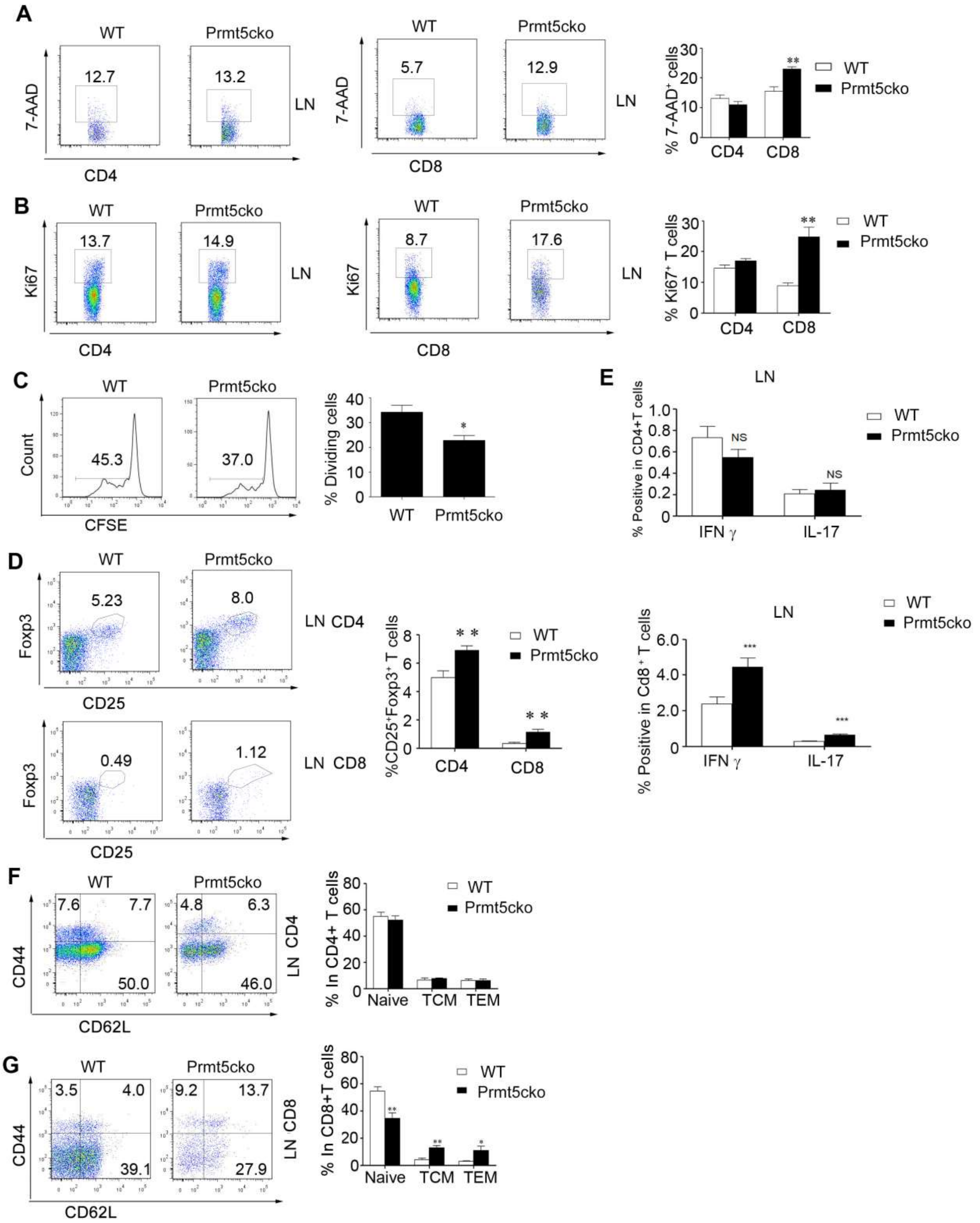

Fig. S2. Prmt5 deficiency in T cells showed much more activity in periphery during EAE. Prmt5cko and WT mice (6 mice per group) were induced EAE by MOG35–55/CFA immunization, at the peak of day 17, LN and spleens were acquired. **(A)** The percentages of CD4+T and CD8+T dead cells in the two groups from LN. (**B**) Percentages of CD4+T and CD8+ proliferation cells in the two groups from LN. (**C**)

CD4+T cells were isolated from spleen and stimulated with MOG for 72h, percentage of dividing cells was determined. (**D**) Percentages of Foxp3+CD25+ Tregs in CD4+ and CD8+ T cells from LN between the two groups. (**E**) Percentages of CD8+Il-17+ and CD8+IFNγ+ T cells from LN between the two groups. (**F, G**) Percentages of CD62L+CD44- (Naïve), CD62L+CD44+ (CM), and CD62L-CD44+ (EM) T cell subsets in CD4+T (**F**), CD8+T (**G**) from LN between the two groups. Data are shown as the mean ± SEM of indicated number of samples and are from a single experiment representative of three experiments performed. Statistical differences were determined by the two-tailed unpaired Student's t test, *$P < 0.05$, **$P < 0.01$, ***$P < 0.01$, NS: not significant.

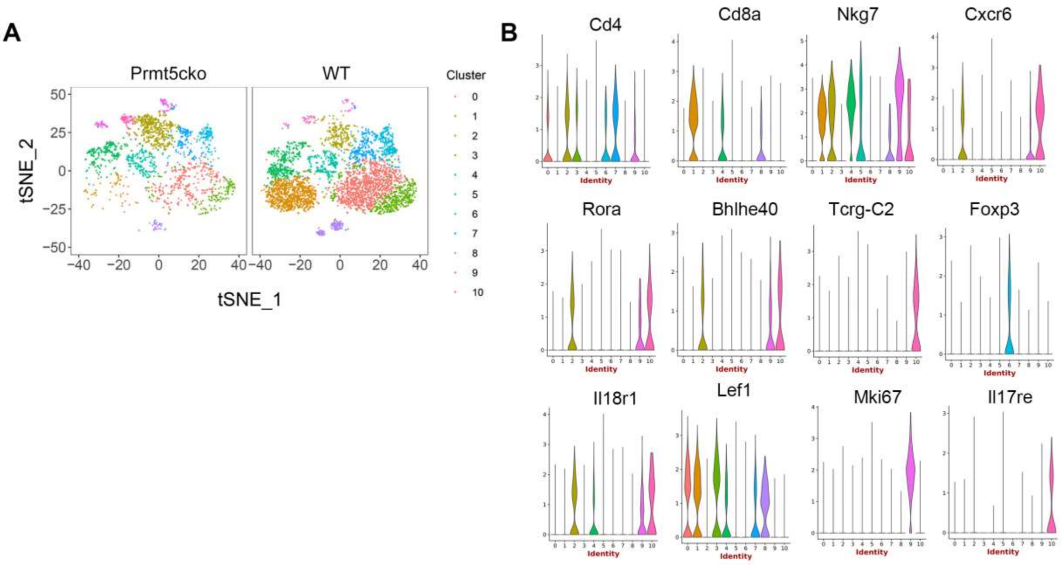

Fig. S3. Single-cell RNA sequencing data related to Figure 5. Prmt5cko and WT mice (3 mice per group) were induced EAE by MOG35–55/CFA immunization, at the peak of day 17, spleen cells were isolated and were performed single cell RNA sequencing. (**A**) tSNE plot showed the spleen cells clusters between these two groups. Each dot corresponds to a single cell, colored according to the cell cluster. (**B**) Violin plot showed the expression levels of relative marker genes，expression was measured as the log2 (count+1).

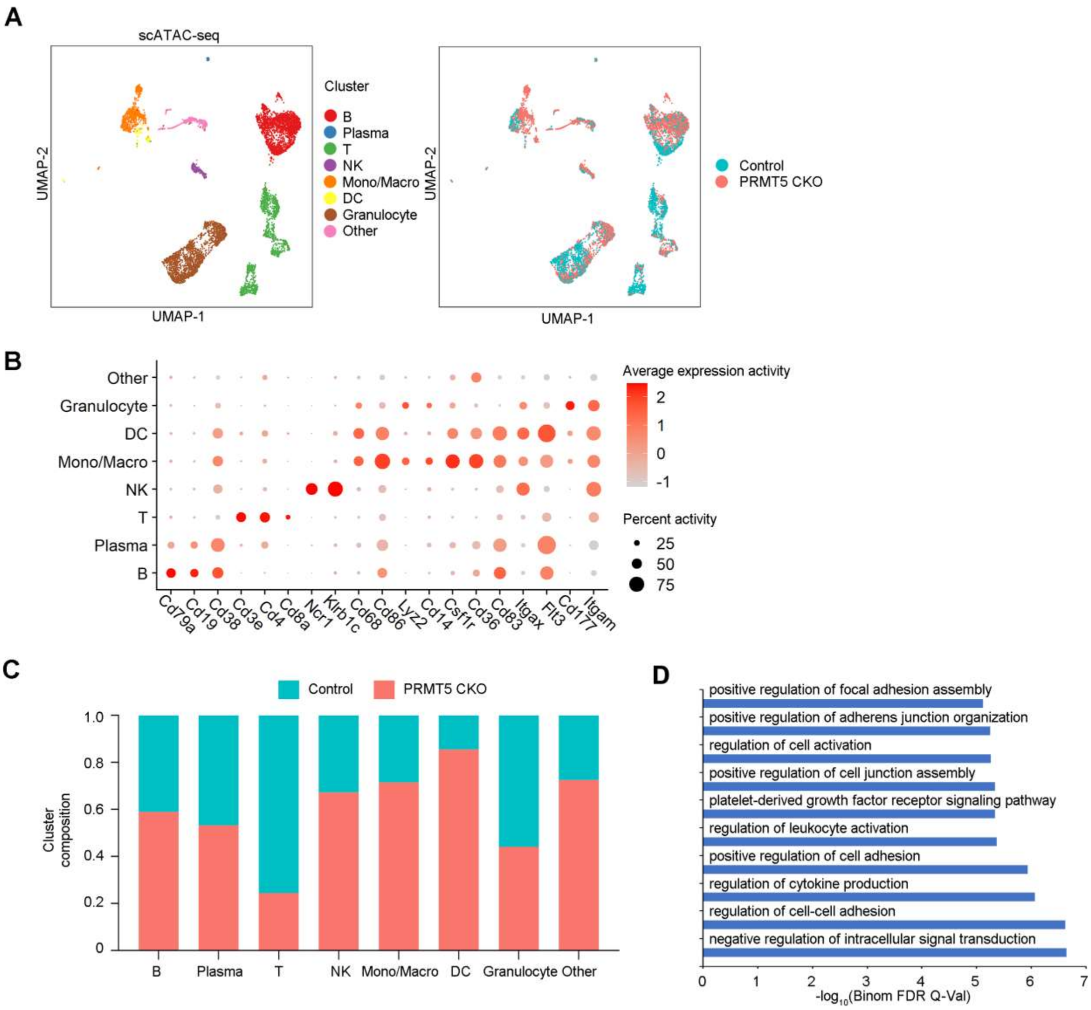

Fig. S4. Single-cell ATAC sequencing data related to Figure 6.

Prmt5cko and WT mice (3 mice per group) were induced EAE by MOG35–55/CFA immunization, at the peak of day 17, spleen cells were isolated and scATAC-seq was performed. (**A**) scATAC-seq clusters of 7518 spleen cells pooled from WT and Prmt5cko mice. (**B**) Summary of gene activity used to identify cell clusters. (**C**) Cluster composition of scATAC-seq clusters between WT and Prmt5cko mice spleen cells. (**D**) GO enrichment analysis of significantly downregulated peaks of CD4-C3-Rora cells between WT and Prmt5cko mice.

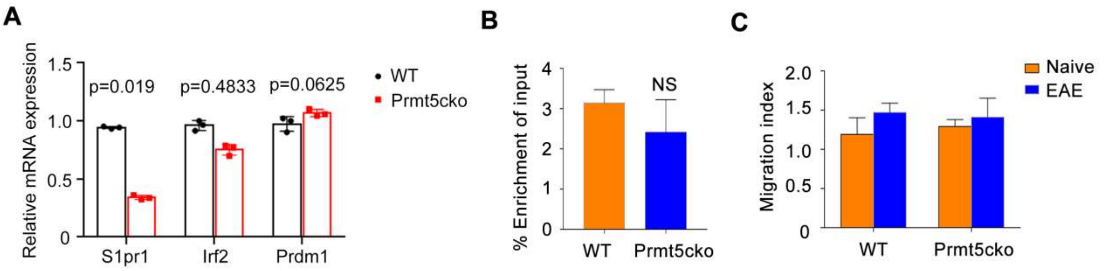

Fig. S5. Irf2 and Prdm1showed no difference between the Prmt5cko and WT mice.

(**A**) EAE was induced in Prmt5cko and WT mice (3 mice per group) by MOG35–55/CFA immunization, and at the peak of Day 17, S1pr1, Irf2 and Prdm1 expressions were measured by RT–qPCR in CD4+T cells. (**B**) Enrichment of H4R3e2s at the S1pr1 promoter was assessed by ChIP Q-PCR in CD4+T cells isolated from Prmt5cko and WT groups in the EAE condition. (**C**) EAE was induced in Prmt5cko and WT mice by MOG35–55/CFA immunization, and at the peak of Day 17, splenocytes were acquired from WT and Prmt5cko mice with or without the EAE induction, and CD3+CD4- T cells migration ability was stimulated with S1P and the cells numbers were measured counted by flow cytometry, the Mmigration index = (S1P group count) / (control group count) was calculated between the groups. Data are shown as the mean ± SEM of indicated number of samples and are from a single experiment representative of two experiments performed. Statistical differences were determined by the two-tailed unpaired Student's t test, NS: not significant.
